# Malate valves: old shuttles with new perspectives

**DOI:** 10.1111/plb.12869

**Published:** 2018-07-17

**Authors:** J. Selinski, R. Scheibe

**Affiliations:** ^1^ Department of Animal, Plant, and Soil Science Australian Research Council Centre of Excellence in Plant Energy Biology School of Life Science La Trobe University Bundoora Bundoora Australia; ^2^ Division of Plant Physiology Department of Biology/Chemistry University of Osnabrueck Osnabrueck Germany

**Keywords:** Energy supply, malate dehydrogenase, malate valve, redox balance, shuttling

## Abstract

Malate valves act as powerful systems for balancing the ATP/NAD(P)H ratio required in various subcellular compartments in plant cells. As components of malate valves, isoforms of malate dehydrogenases (MDHs) and dicarboxylate translocators catalyse the reversible interconversion of malate and oxaloacetate and their transport. Depending on the co‐enzyme specificity of the MDH isoforms, either NADH or NADPH can be transported indirectly. *Arabidopsis thaliana* possesses nine genes encoding MDH isoenzymes. Activities of NAD‐dependent MDHs have been detected in mitochondria, peroxisomes, cytosol and plastids. In addition, chloroplasts possess a NADP‐dependent MDH isoform. The NADP‐MDH as part of the ‘light malate valve’ plays an important role as a poising mechanism to adjust the ATP/NADPH ratio in the stroma. Its activity is strictly regulated by post‐translational redox‐modification mediated *via* the ferredoxin‐thioredoxin system and fine control *via* the NADP ^+^/NADP(H) ratio, thereby maintaining redox homeostasis under changing conditions. In contrast, the plastid NAD‐MDH (‘dark malate valve’) is constitutively active and its lack leads to failure in early embryo development. While redox regulation of the main cytosolic MDH isoform has been shown, knowledge about regulation of the other two cytosolic MDHs as well as NAD‐MDH isoforms from peroxisomes and mitochondria is still lacking. Knockout mutants lacking the isoforms from chloroplasts, mitochondria and peroxisomes have been characterised, but not much is known about cytosolic NAD‐MDH isoforms and their role *in planta*. This review updates the current knowledge on MDH isoforms and the shuttle systems for intercompartmental dicarboxylate exchange, focusing on the various metabolic functions of these valves.

## Introduction

The redox state provides information on the metabolic status of a plant cell, which is needed for fast adjustment of various metabolic activities in response to continuously changing environmental conditions and developmental requirements (Dietz 2003; Foyer & Noctor 2003; Scheibe *et al*. 2005). Since major imbalances in the redox state can cause severe damage, plant cells possess different redox sensors such as proteins with reversibly oxidisable thiols (*e.g*. cytosolic glyceraldehyde‐3‐phosphate dehydrogenase, cyclophilin 20‐3) which detect deviations from redox homeostasis and activate poising mechanisms, thus avoiding the generation of excess reactive oxygen species (ROS; Scheibe & Dietz 2012; Park *et al*. 2013; Hildebrandt *et al*. 2015; Noctor & Foyer 2016).

Chloroplasts possess a light‐dependent regulatory mechanism, namely the ferredoxin‐thioredoxin system (Fd‐Trx system), controlling the activities of various key enzymes involved in assimilatory and dissimilatory pathways (Buchanan 1984; Buchanan & Balmer 2005). Reduced Trx subsequently reduces regulatory disulfide bonds of various target enzymes, thereby altering their catalytic activities. Upon illumination, various Calvin‐Benson cycle (CBC) enzymes are activated *via* the Fd‐Trx system, whereas oxidative pentose phosphate pathway (OPPP) enzymes, such as glucose‐6‐phosphate dehydrogenase, become inactive. The light/dark modulation of various chloroplast proteins *via* the Fd‐Trx system functions to control synthesis and degradation of carbohydrates to prevent futile cycling and waste of energy, by adjusting enzyme activities at the post‐translational level.

The regulation of energy metabolism is particularly complex in plants. On the one hand, ATP and NAD(P)H are generated in a number of reactions that are localised in different subcellular compartments. During illumination, photosynthesis and respiration generate the energy carrier ATP and the reducing equivalents NADPH and NADH in chloroplasts, cytosol and mitochondria, respectively, in green parts of the plant, whereas glycolysis, respiration and the OPPP produce these energy molecules in the dark and in non‐green tissues in various compartments. In turn, NAD(P)H and ATP are required for all energy‐consuming reactions, such as assimilation of C, N, S and all other cellular activities, both during photoautotrophic and heterotrophic growth. However, the delivery of NAD(P)H and ATP for various energy‐consuming reactions should occur at the required rates and in the specific compartments due to the rather limited pool sizes of these energy sources (Scheibe 2004).

**Figure 1:**
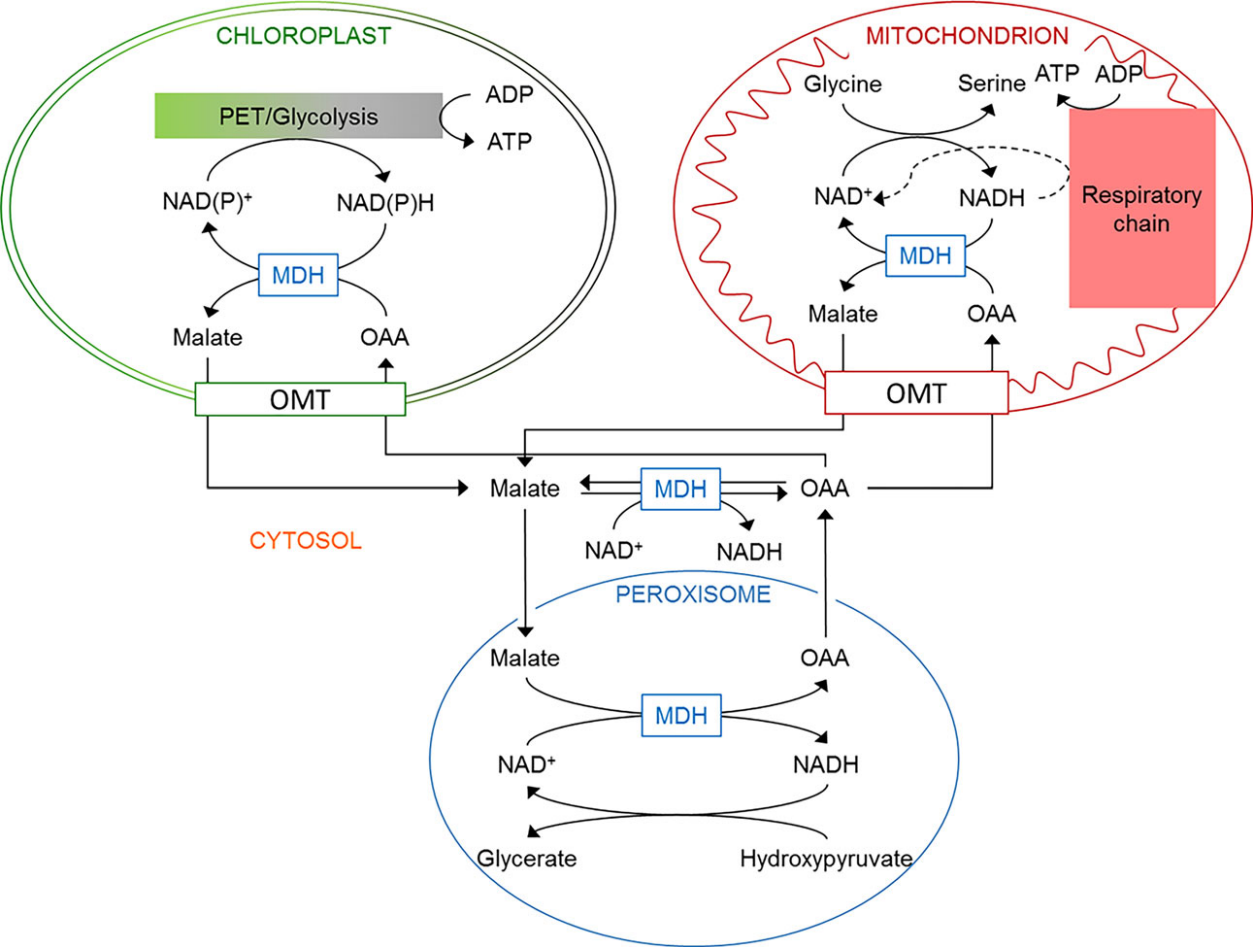
Localisation of malate dehydrogenase isoforms. Malate/OAA translocators together with malate dehydrogenases (malate valves) enable the indirect transfer of reducing equivalents between different subcellular compartments in plant cells. MDH, malate dehydrogenase; OAA, oxaloacetate; PET, photosynthetic electron transport; OMT, malate/OAA translocators.

Plant membranes are essentially impermeable to NAD(P)^+^ and NAD(P)H. Therefore, plant cells possess specific translocators enabling the direct and indirect exchange of reducing equivalents. On the one hand, NAD(P)^+^ can directly be shuttled between subcellular compartments *via* NAD^+^‐carrier proteins localised in mitochondria as well as in plastids (Palmieri *et al*. 2009). However, these transporters exhibit a low affinity for NADH and NADPH (Palmieri *et al*. 2009; Maurino & Engqvist 2015). On the other hand, plant cells possess specific translocators for the exchange of dicarboxylates such as malate and oxaloacetate (malate/OAA shuttles; Fig. 1). Malate is a versatile compound in plant metabolism (Lance & Rustin 1984; Maurino & Engqvist 2015) that can easily be transported across subcellular membranes and can be used as a substrate for mitochondrial ATP production or provision of NADH to the cytosol (Fig. 1). Malate/OAA shuttles acting in combination with malate dehydrogenases (MDHs) as key enzymes (also termed malate valves) connect compartments. They are powerful systems for balancing metabolic fluxes by enabling indirect transfer of reducing equivalents, and therefore, they play a key role in plant metabolism.

### A multigene family encodes malate dehydrogenases in *Arabidopsis*

The NAD‐dependent MDH activities have been detected in chloroplasts, mitochondria and peroxisomes, as well as in the cytosol (Gietl 1992; Berkemeyer *et al*. 1998). Besides the non‐redox regulated NAD‐MDH, chloroplasts of higher plants and green algae additionally contain a redox‐modulated NADP‐dependent MDH (Scheibe & Anderson 1981).

**Figure 2:**
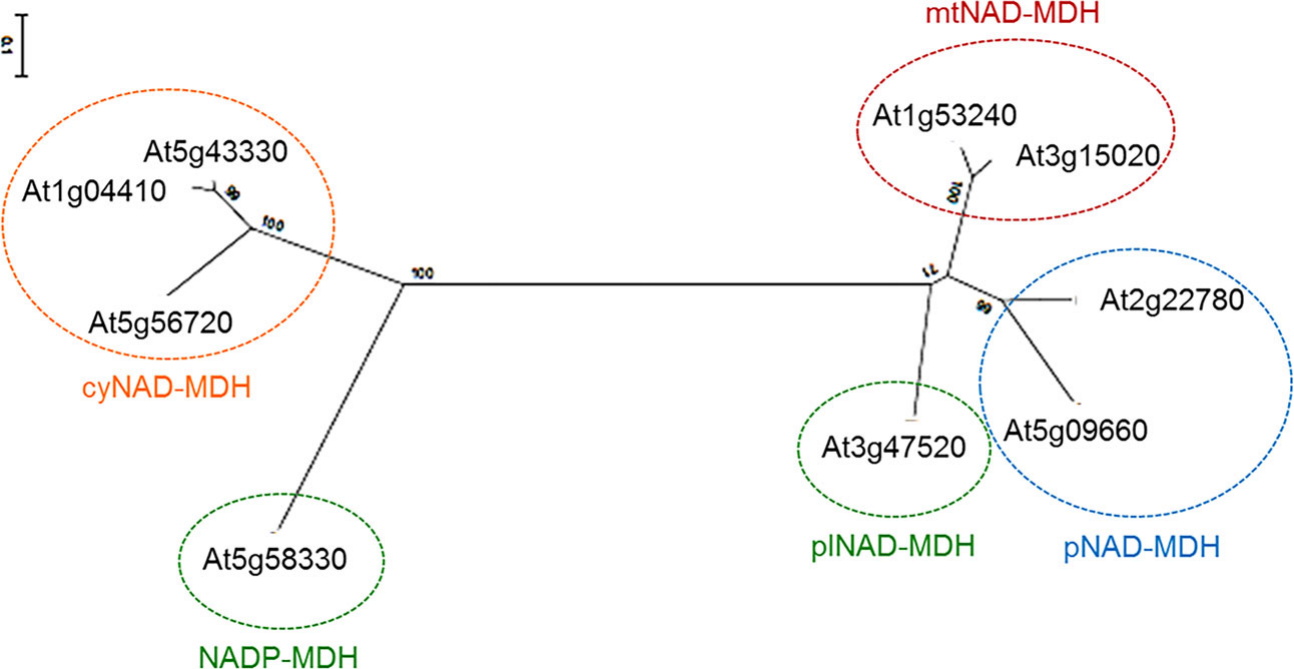
Phylogenetic tree of MDH isoforms from *A. thaliana*. Amino acid sequences encoding the mature MDH isoproteins were aligned *via* the Genedoc tool (updated version from 2007; Nicholas *et al*. 1997). The phylogenetic tree was calculated with the program MEGA 6 (Tamura *et al*. 2013) using the neighbour‐joining tree method and the statistical approach of bootstrapping. Bootstrap replications were set to 1000. cyNAD‐MDH, cytosolic malate dehydrogenase (cyNAD‐MDH 1, At1g04410; cyNAD‐MDH 2, At5g43330; cyNAD‐MDH 3, At5g56720); pNAD‐MDH, peroxisomal malate dehydrogenase (pNAD‐MDH 1, At2g22780; pNAD‐MDH 2, At5g09660); mtNAD‐MDH, mitochondrial malate dehydrogenase (mtNAD‐MDH 1, At1g53240; mtNAD‐MDH 2, At3g15020); plNAD‐MDH, plastidic malate dehydrogenase (At3g47520); NADP‐MDH, chloroplast malate dehydrogenase (At5g58330).

All eukaryotic MDH isoforms can be traced back to two ancestral genes (Ocheretina & Scheibe 1997; Ocheretina *et al*. 2000). One of the two ancestral genes represents the origin of cytosolic NAD‐dependent MDH isoenzymes (cyNAD‐MDH 1–3) and the NADP‐MDH in chloroplasts (Fig. 2). These isoforms arose through duplication of the ancestral gene. The second subfamily includes NAD‐MDHs of mitochondria (mtNAD‐MDH 1–2), plastids (plNAD‐MDH) and peroxisomes (pNAD‐MDH 1–2), which resulted from triplication of a pre‐existing mitochondrial MDH gene and major changes within their target sequences (Fig. 2).

Within each subfamily the amino acid identity of the mature proteins is above 50%. Between the two subfamilies, amino acid identity lies at about 21–23%. However, MDHs possess similar tertiary and quaternary structures and almost identical catalytic properties (McAlister‐Henn 1988). Each precursor protein of the organellar MDH isoforms possesses a characteristic N‐terminal transit peptide (Gietl & Hock 1982). For instance, the plNAD‐MDH from *Arabidopsis* was originally identified by its unique transit peptide of 80 amino acids despite the high identity with coding sequences of the cytosolic isoforms (Berkemeyer *et al*. 1998; Imsande *et al*. 2001).

### The light malate valve in chloroplasts is strictly regulated

The NADP‐MDH is strictly regulated by light through the Fd‐Trx system. The NADP‐MDH is present as a homodimer which possesses eight cysteine residues per subunit. Two cysteine pairs are located in the N‐ and C‐terminal extensions, whereas the other four cysteine residues are present in the core of the protein (Crétin *et al*. 1990). The redox‐sensitive cysteine pairs in the N‐ and C‐terminal regions are involved in the light‐dependent redox modulation of the NADP‐MDH *via* the Fd‐Trx system during photosynthesis; upon removal of one or the other sequence extension, the enzyme is constantly active (Fickenscher & Scheibe 1988; Ocheretina *et al*. 1993). This indicates that removal of the regulatory structures of the conserved globular MDH protein does not affect its activity but rather has evolved to allow for control (Scheibe 1991).

Under oxidising conditions, the N‐terminal extension of the NADP‐MDH is involved in dimerisation linking the subunits by hydrophobic interactions. In addition, the C‐terminal disulfide pulls the C‐terminal extension into the active site, blocking the place for the substrate, hence acting as an internal inhibitor of the protein. Upon reduction by the Fd‐Trx system, the C‐terminal extension of NADP‐MDH is released from the active centre while the reduction of the N‐terminal extension results in a more relaxed structure of the active site. In a two‐step reaction, finally the fully active enzyme is formed (Miginiac‐Maslow *et al*. 1997, 2000).

In the light, a continuous reduction by the Fd‐Trx system and the simultaneous re‐oxidation of the target enzymes by O_2_ allows for adjustment of the activation state of the target enzymes also in the light, since this cycling allows for flexible adjustment of the ratio between reduced to oxidised form. These redox cycles represent the basis for the fine control of enzyme activities. Activation and inactivation rates of the target enzymes are influenced by specific effectors, mainly substrates and products of the respective target enzyme, acting as a positive or negative effector (Heineke & Scheibe 2009). For instance, the activation of NADP‐MDH is only enabled when NADPH increases because it is not consumed in the CBC, *e.g*. due to a lack of CO_2_ or ATP. Under these conditions, the NADP‐MDH uses the excess NADPH generated *via* the photosynthetic electron transport chain to convert OAA to malate, regenerating the electron acceptor NADP^+^. An increased level of NADP^+^ in turn acts as a negative effector for reductive activation of the NADP‐MDH, in this way turning down its activity. This fine control guarantees that reducing equivalents are only exported to other subcellular compartments *via* the malate/OAA shuttle when they are in excess, and when they are not required in the chloroplast for assimilatory processes (Scheibe 1987, 1991; Backhausen *et al*. 2000). Upon a sudden increase in light intensity, NADP‐MDH activity rapidly increases within less than a minute and subsequently oscillates until the new steady‐state is reached (Scheibe & Stitt 1988). Such hysteretic response is characteristic for this type of post‐translational regulation.

Although NADP‐MDH is known to function as a poising mechanism avoiding the generation of ROS under high light, *nadp‐mdh* knockout mutants are indistinguishable from the wild type under various stress conditions when grown in soil. This is due to alternative systems such as the NADPH‐dependent thioredoxin reductase (NTRC)/2‐Cys‐peroxiredoxin system, as well as proline biosynthesis, photorespiration and nitrate assimilation in *nadp‐mdh* knock‐out mutants contributing to the maintenance of redox homeostasis when malate valve capacities are lacking (Hebbelmann *et al*. 2012; Selinski & Scheibe 2014).

Upon sustained high‐light intensity, an increase in the expression level of the nuclear encoded NADP‐MDH points to a signal transfer from the chloroplast to the nucleus (Becker *et al*. 2006). The nature of such a signal is still not yet clarified, but a transient increase in H_2_O_2_ has been put forward as a potential signal since plants lacking NADP‐MDH do not show such an increase (Heyno *et al*. 2014). Transfer of the signal might occur *via* oxidative modification of GapC (Hildebrandt *et al*. 2015). The glycolytic GapC, possibly in its ‘moonlighting’ function, had been found to bind to the NADP‐MDH gene (Hameister *et al*. 2007).

It is conceivable that *in vivo* kinetic effects override the thermodynamic equilibrium of the NADP‐MDH reaction. In particular, when fluctuating conditions give rise to immediate deviations from steady state. Then high pressure on the side of influx, *e.g*. light intensity, shifts the equilibrium towards efflux, namely the reduction of OAA. Literally, the excess electrons are then pushed through the malate valve. Malate is used for further reactions outside the chloroplasts where various options are possible, in the worst case, the dissipation of energy in the alternative oxidase pathway in the mitochondria. The flux of reductant through the valve will continue as long as the pressure from inside the chloroplast exists and keep it open. Such control is achieved by fine‐tuning of NADP‐MDH activation state by the NADP^+^/NADP(H) ratio in the chloroplast, with NADP^+^ hindering activation.

Factors that sustain a steady homeostatic flux through the CBC also include the controlled export of reducing equivalents from the chloroplast in the form of malate as required for balancing the ATP/NADPH ratio (Fridlyand *et al*. 1998; Fridlyand & Scheibe 1999). Due to the rapid responses of redox‐regulated enzymes, such as NADP‐GAPDH, NADP‐MDH and others, to changing metabolite levels, homeostasis is maintained in a stable non‐equilibrium state by thermodynamic buffering (Igamberdiev & Kleczkowski 2011). The system is kept in such a stable and, at the same time, flexible state as long as energy from light comes in and product is taken out. Even reactions close to the thermodynamic equilibrium are able to limit homeostatic fluxes when their total capacities are decreased in transgenic plants (Fridlyand & Scheibe 2000). Using the kinetic data available for the fine‐control of NADP‐MDH, a model has been developed that supports the function of this enzyme to control the indirect export of NADPH (Fridlyand *et al*. 1998).

### The dark malate valve in plastids is constitutively active

In addition to the NADP‐dependent MDH, chloroplasts contain a redox‐independent activity of NAD‐MDH functioning as part of the dark malate valve and playing an important role in dark metabolism. Non‐green plastids also contain such plNAD‐MDH. In these plastids and in chloroplasts during darkness, ATP and NADPH for anabolism are generated independently *via* plastidial glycolysis (ATP), and plastidial OPPP (NADPH). The dark malate valve is here required to export the NADH that is formed during glycolysis at the substrate phosphorylation step catalysed by PGK and GAPDH. NADH production in this step occurs by either the bispecific CBC enzyme GapA/B in chloroplasts (Baalmann *et al*. 1995) or GapCp in plastids of non‐green tissues (Backhausen *et al*. 1998; Muñoz‐Bertomeu *et al*. 2009, 2010). Such oxidation of triose phosphates coupled with ATP generation requires a continuous supply of NAD^+^. The regeneration of NAD^+^ which allows for continued ATP production *via* glycolysis is possible due to the activity of plNAD‐MDH for indirect export of NADH in the form of malate. In addition, ATP can be imported into plastids *via* ATP/ADP transporters of the NTT type (Kampfenkel *et al*. 1995; Neuhaus *et al*. 1997; Möhlmann *et al*. 1998; Tjaden *et al*. 1998; Reiser *et al*. 2004). However, although these ATP/ADP transporters exhibit high affinities for ATP and ADP, their capacities are low (*V*_max_ for ATP (NTT1) = 24 nmol·mg protein^−1^·h^−1^; *V*_max_ for ATP (NTT2) = 7 nmol·mg protein^−1^·h^−1^; Möhlmann *et al*. 1998). Therefore, an indirect mechanism for ATP import in plastids is more favourable, *e.g. via* shuttling of glycolytic intermediates such as glucose 6‐phosphate (G‐6‐P) or phosphoenolpyruvate (PEP). These intermediates can be transported from the cytosol into plastids or *vice versa via* G‐6‐P/P_i_ translocators or PEP/P_i_ translocators (Facchinelli & Weber 2011). Homozygous *plnad‐mdh* knock‐out mutants do not exist. This is either due to compromised male or female gametophyte development (pollen or egg cell) in haploid stages of the plant or impaired embryo development and seed maturation in diploid stages. A total knock‐out of plNAD‐MDH that is present in 50% of the haploid pollen grains of heterozygous *plnad‐mdh/plNAD‐MDH* mutants causes impaired pollen tube growth *in vitro*, which can be overcome by adding substrates of NADH‐dependent glutamine‐2‐oxoglutarate aminotransferase (GOGAT; Selinski *et al*. 2014). However, pollen tube growth does not appear to be affected *in vivo*. This indicates that the maternal tissue in the transducing tract of the style can supply the NADH‐GOGAT substrates, in this way enabling pollen tube elongation. Although NADH‐GOGAT was shown to be an alternative pathway maintaining redox homeostasis in heterozygous *plnad‐mdh/plNAD‐MDH* plants and *plnad‐mdh* knock‐out pollen when the capacity of the dark malate valve is lacking, NADH‐GOGAT cannot compensate for the homozygous knock‐out of *plnad‐mdh* during embryogenesis leading to an embryo‐lethal phenotype (Beeler *et al*. 2014; Selinski *et al*. 2014). Heterozygous *plnad‐mdh/plNAD‐MDH* mutants are able to grow, flower and set seed, but their siliques contain green as well as white seeds during the ripening process. Microscopic analysis of green and white seeds revealed that green seeds contain developed embryos at all developmental stages whereas white seeds exclusively contain embryos in the globular stage, independent of the developmental stage of the silique. These white seeds develop into tiny, shrunken and non‐vital seeds that can easily be lost during seed cleaning procedures. Accordingly, the ratio of green to white seeds was determined as 3:1, which correlates with the theoretical ratio of 1:2:1 for wild types (green seeds) to heterozygotes (green seeds) to homozygotes (white seeds) after selfing.

The lack of chlorophyll in homozygous *plnad‐md*h knock‐out seeds might be due to competition for glutamate, which is the precursor for chlorophyll biosynthesis as well as the substrate for the chloroplast 2‐oxoglutarate (2‐OG)/malate transporter AtOMT1 together with the dicarboxylate transporter DCT1. OMT1 and DCT1 play important roles in both the malate valve and the 2‐OG/malate shuttle for nitrate assimilation (Taniguchi *et al*. 2002; Kinoshita *et al*. 2011). Glutamine and 2‐OG must be provided to the plastid in exchange for glutamate to support the NADH‐GOGAT step (Fig. 4). When plNAD‐MDH is lacking, maintenance of redox homeostasis and ATP production *via* glycolysis then depend on the substrate glutamate. Although an increased flux through the NADH‐GOGAT pathway maintains redox homeostasis when plNAD‐MDH is lacking in homozygous progeny of the non‐lethal heterozygous mutant line, this increased flux probably leads to impaired chlorophyll biosynthesis, stopping embryo development at the globular stage (Selinski *et al*. 2014).

### Cytosolic MDHs mediate intercompartmental energy exchange

As described above, excess reducing equivalents from the chloroplast are indirectly transferred as malate to the cytosol and to other subcellular compartments to avoid imbalances or lack of electron acceptors that would lead to oxidative stress (Scheibe 2004). The interconversion of malate to OAA with concomitant reduction of NAD^+^ to NADH can provide reducing equivalents for nitrate reduction in the cytosol or ATP from mitochondrial respiration *via* oxidative phosphorylation. If neither NADH nor ATP are required, energy dissipation *via* the alternative respiratory pathway consisting of alternative NAD(P)H dehydrogenases and the alternative oxidase (AOX) can serve to protect the cell from oxidative stress (Fig. 4). Hence, cyNAD‐MDH functions as a key player in the transfer of reducing equivalents from the chloroplast to other sinks in plant cells, and even allows for conversion of reducing equivalents into ATP or, in the worst case, energy dissipation as heat *via* AOX.

In *Arabidopsis*, three NAD‐MDH isoforms are localised in the cytosol. So far, only the main isoform (cyNAD‐MDH 1) has been well characterised and described. Using the recombinant enzyme, cyNAD‐MDH 1 is active under reducing conditions, while oxidising conditions lead to its inactivation (Hara *et al*. 2006). Apparently, this isoform is sensitive to H_2_O_2_ through sulphur oxidation of cysteines and methionines affecting its kinetics, secondary structure and thermodynamic stability. Furthermore, the reversible homodimerisation and reduction of cyNAD‐MDH 1 protects the protein from over‐oxidation (Huang *et al*. 2018).

### Peroxisomal MDHs are involved in photorespiration and in fatty acid β‐oxidation

Leaf peroxisomes and glyoxysomes in lipid‐storing tissues are the two main types of microbodies in plant cells. Peroxisomes rely on reducing equivalents imported from mitochondria or chloroplasts by the malate‐OAA shuttles, since they do not possess any metabolic pathways capable of delivering NADH at the high rates required *e.g*. for photorespiration. Reducing equivalents are imported into peroxisomes through the uptake of malate and its conversion to OAA by pNAD‐MDH. In glyoxysomes, the pNAD‐MDH is essential for fatty acid β‐oxidation in germinating *A. thaliana* seeds. In leaf peroxisomes, it is needed to maintain optimal rates of photorespiration by providing NADH required for the reduction of hydroxypyruvate to glycerate by peroxisomal hydroxypyruvate reductase (HPR; Cousins *et al*. 2008; Pracharoenwattana *et al*. 2010; Timm *et al*. 2011). Glycerate can be transported back to the chloroplast where it is phosphorylated to yield 3‐phosphoglycerate, which can re‐enter the CBC (Ogren 1984; Leegood *et al*. 1995; Boldt *et al*. 2005; Bartsch *et al*. 2010; Walker *et al*. 2016).

### Mitochondrial MDHs play an important role in photorespiration and the provision of NADH for respiration

Photorespiration results in NADH formation in plant mitochondria due to the oxidation of glycine to serine by the glycine decarboxylase complex (GDC). NADH can enter the respiratory electron transport chain, supporting ATP synthesis. Coupling of malate oxidation by mtNAD‐MDH with GDC and the pyruvate dehydrogenase complex (PDC) reaction, respectively, in mitochondria helps to maintain high fluxes, apparently by recycling NAD^+^ required as electron acceptors for decarboxylating glycine and pyruvate oxidation in both complexes (Lindén *et al*. 2016). It has been discussed that mtNAD‐MDH could play an important role in decreasing the concentration of NADH and relieving its inhibitory effect on PDC (Igamberdiev *et al*. 2014), or mtNAD‐MDH could recycle the NADH from glycine oxidation to keep NADH concentrations low and thus prevent substrate inhibition of GDC (Bykova *et al*. 2014).

Furthermore, mtNAD‐MDH was found to lower leaf respiration, probably based on cross‐talk between mtNAD‐MDH and the cytochrome *bc*_*1*_ complex (Wang *et al*. 2010). It also appears to alter the rate of photorespiration as well as plant development, demonstrating its important role in energy metabolism and redox homeostasis (Tomaz *et al*. 2010; Wang *et al*. 2010; Lindén *et al*. 2016). Proteomic analyses revealed that mtNAD‐MDH 1 significantly accumulates during *Arabidopsis* seed germination (Fu *et al*. 2005), and transcript abundance of both mtNAD‐MDH isoforms significantly increases in early stages of seed germination (Weitbrecht *et al*. 2011). In accordance with analyses of plNAD‐MDH mutants, mtNAD‐MDH was shown to play an important role in the earliest phases of the *Arabidopsis* life cycle (Beeler *et al*. 2014; Selinski *et al*. 2014; Sew *et al*. 2016). Recently, mtNAD‐MDH 1 was shown to be insensitive to thiol‐based redox control (*in vitro* and *in vivo*) but its activity is lowered by adenine nucleotides, especially ATP (Yoshida & Hisabori 2016). Mitochondrial NAD‐MDH was demonstrated to play a significant role in buffering the redox state in the mitochondrial matrix due to its equilibrium properties (Hagedorn *et al*. 2004). For any calculation of *in vivo* concentrations of NADH, NAD^+^ and metabolite concentrations, it is essential to take into account the fact of ‘molecular crowding’ in the cell, resulting in binding of ‘small molecules’ to proteins and to changed concentrations of free soluble compounds (Srere 1980). In an update on the current approaches for metabolic flux analyses, factors are compiled that determine the specific properties of a metabolic network as far as its stability and capability to maintain homeostasis is concerned (Nikoloski *et al*. 2015). In particular, the authors point out that more data are required so that the interrelationship of metabolite levels, fluxes and enzyme properties can be integrated into mathematical models.

### Plant cells possesses various dicarboxylate transporters in chloroplasts and mitochondria

The compartmentation of plant cells requires the transport of metabolites across intracellular membranes catalysed by specific membrane translocators. Especially, the inner membranes of chloroplasts and mitochondria represent the permeability barrier for metabolites and co‐enzymes and are equipped with various transporters that are specific for the various substrates. The *Arabidopsis* genome encodes a number of dicarboxylate transporters catalysing the transfer of dicarboxylates across the chloroplastidic or mitochondrial membranes (Heineke *et al*. 1991; Weber *et al*. 2004; Weber & Fischer 2007; Linka & Weber 2010; Facchinelli & Weber 2011; Fischer 2011; Flügge *et al*. 2011; Haferkamp & Schmitz‐Esser 2012; Lee & Millar 2016).

**Figure 3:**
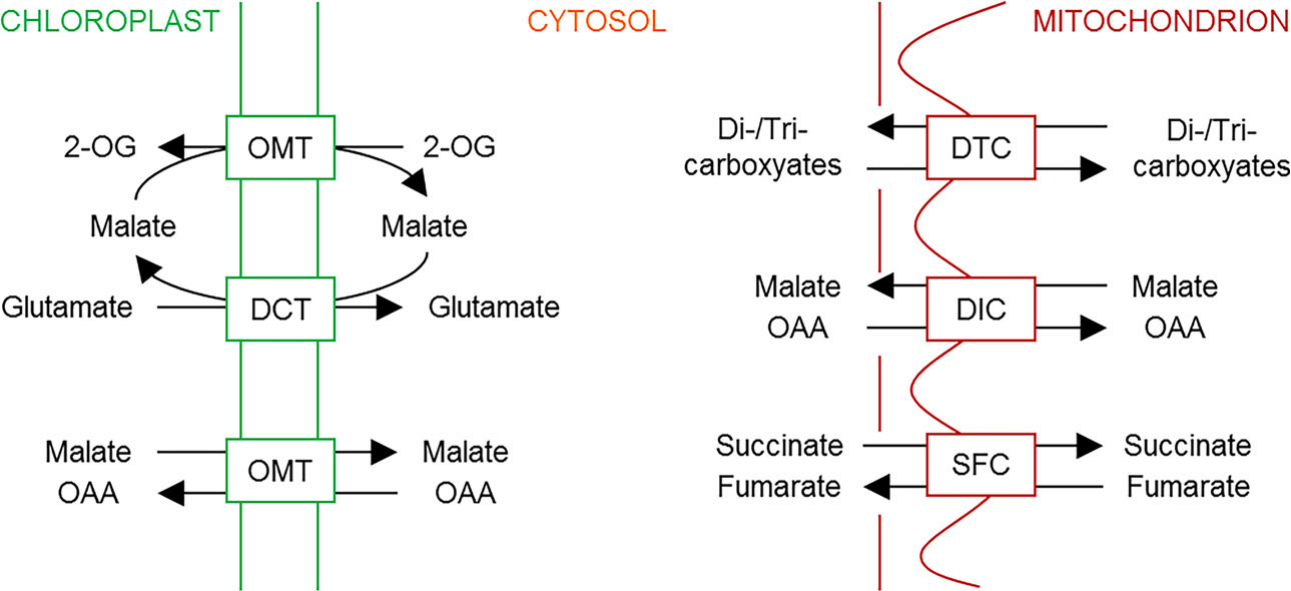
Shuttles for dicarboxylate transport. Transport processes of dicarboxylates across the inner envelope of plastids and the inner mitochondrial membrane are depicted. 2‐OG, 2‐oxoglutarate; DCT (= DiT2), glutamate/malate translocator; DIC, decarboxylate carriers; DTC, dicarboxylate/tricarboxylate carrier; OAA, oxaloacetate; OMT (= DiT1), 2‐OG/malate‐translocator, malate/OAA translocator; SFC, succinate/fumarate carrier.

The two plastid‐localised dicarboxylate translocators (DiTs) are involved in primary ammonium assimilation and the recycling of ammonium that is lost during photorespiration. DiT1 (also termed OMT1, 2‐OG/malate‐translocator) imports the precursor of ammonium assimilation, 2‐OG, in exchange for malate. DiT2 (also termed DCT, glutamate/malate‐translocator) exports glutamate in exchange for malate (Fig. 3; Weber *et al*. 1995; Weber & Flügge 2002; Renne *et al*. 2003; Schneidereit *et al*. 2006; Riebeseel *et al*. 2010). In addition to its high affinity for 2‐OG and malate, DiT1 displays a high affinity for OAA and has been proposed as the OAA/malate valve (Taniguchi *et al*. 2002). The role as malate valve for DiT1 has been proven by mutant analyses, showing that cytosolic OAA is efficiently transported into chloroplasts mainly by DiT1 (Fig. 3; Kinoshita *et al*. 2011; Taniguchi & Miyake 2012).

In parallel to plastids, mitochondria contain various dicarboxylate translocators. The transport of specific dicarboxylates and tricarboxylates across the inner mitochondrial membrane is required for several metabolic processes, such as primary amino acid synthesis, export of reducing equivalents, fatty acid metabolism, gluconeogenesis and isoprenoid biosynthesis. The plant mitochondrial dicarboxylate/tricarboxylate carrier (DTC) mediates the transport of dicarboxylates (2‐OG, OAA, malate and succinate) and tricarboxylates (citrate, isocitrate and aconitate) by counter‐exchange (Fig. 3; Picault *et al*. 2002). Furthermore, plant mitochondria possess three dicarboxylate carriers (DIC1–3). These transporters facilitate the transfer of dicarboxylates, such as malate, OAA and succinate, as well as phosphate, sulphate and thiosulphate across the inner mitochondrial membrane. Accordingly, DICs may play an important role in the import of dicarboxylates into mitochondria in exchange for phosphate or sulphate to replenish the tricarboxylic acid cycle (TCAC). In addition, DICs might function as malate/OAA shuttles providing other cell compartments with reducing equivalents (Palmieri *et al*. 2008). Another dicarboxylate transporter, SFC, has been identified in plant mitochondria (Fig. 3). This carrier catalyses the transfer of succinate and fumarate across the inner mitochondrial membrane (Catoni *et al*. 2003). The main product of the glyoxylate pathway, succinate, is imported *via* SFC into mitochondria where it can enter the TCAC. In contrast, fumarate is exported to the cytosol in exchange for succinate entering gluconeogenesis after oxidation to malate (Catoni *et al*. 2003).

On both sides of the membranes carrying all of these dicarboxylate shuttle systems, the various MDH isoforms will convert OAA or malate as they arrive on the other side. Due to its equilibrium constant (*K*_eq_ = 2.86 × 10^−5^ at pH 7.0), MDH favours the reaction towards malate formation, *i.e*. the reverse of what is required to regenerate OAA in the mitochondria. Therefore, only removal of the products enables the correct direction for the TCAC. On the other hand, malate is formed in chloroplasts when excess NADPH is used by NADP‐MDH for indirect export of reducing equivalents to the cytosol where it is either reconverted to OAA, when NADH is used for nitrate reduction, or transported to the mitochondria. In both examples, the direction of the enzyme reaction depends on input of substrate and removal of product in a steady‐state situation, but not its equilibrium constant. The transporters generally function as exchange carriers in both directions, driven by the concentration gradient of the respective substrates on both sides. They strictly function as shuttles, however, without any restriction as far as regulation or energy requirements are concerned, only limited by their *V*_max_ and the specificity towards the transported range of substrates. They help to connect the fluxes of energy‐consuming and energy‐converting pathways between the different cellular compartments. Together with the MDH isoforms in each compartment that are operating close to equilibrium, the dicarboxylate translocators support thermodynamic buffering of the cell.

### Assimilation of N is an important backup system maintaining redox homeostasis when plastidial malate valve capacities are lacking or limiting

**Figure 4:**
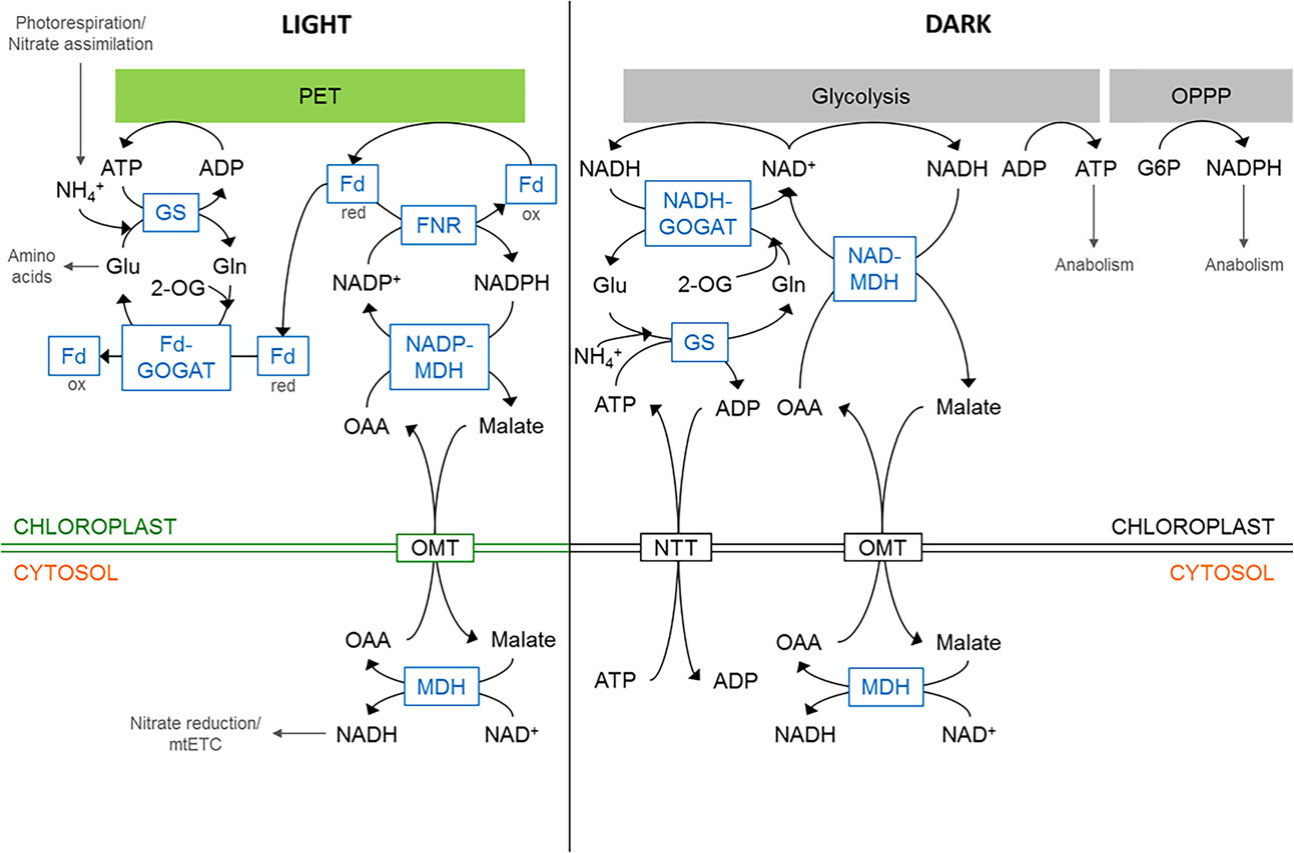
Relationship between plastidial malate valves and NH_4_^+^ assimilation. In the light, Fd‐GOGAT represents an alternative electron acceptor for Fd, and therefore, counteracts accumulation of excess NADPH generated by the Fd‐NADPH reductase (FNR). In the dark, NADH‐GOGAT represents an alternative to plNAD‐MDH, since both are able to regenerate NAD^+^ to maintain continuous ATP production *via* plastidial glycolysis. G6P, glucose 6‐phosphate; Gln, glutamine; Glu, glutamate; GS, glutamine synthetase; mtETC, mitochondrial electron transport chain; NTT, plastidial ATP/ADP transporter; OMT, malate/OAA translocators; OPPP, oxidative pentose phosphate pathway.

The removal of photorespiratory ammonium (NH_4_^+^), which is formed during the oxidation of glycine to serine, is essential for survival of a plant. The primary assimilation of NH_4_^+^ results from the collaborative activity of glutamine synthetase (GS) and GOGAT (GS/GOGAT cycle). GS catalyses the ATP‐dependent assimilation of NH_4_^+^ into glutamine, using glutamate as a substrate. Subsequently, GOGAT catalyses the reductive transfer of the amido group from glutamine to 2‐OG, resulting in the formation of two molecules of glutamate, one of them starting the assimilatory cycle again (Fig. 4). In chloroplasts of higher plants, two major classes of GOGAT enzymes are present, namely the Fd‐dependent and the NADH‐dependent GOGAT, which use either Fd or NADH as a reductant for the interconversion of glutamine and 2‐OG to glutamate (Fig. 4). While NADH‐GOGAT is crucial for NH_4_^+^ assimilation in heterotrophic tissues, Fd‐GOGAT, representing the predominant form in leaves, contributes to the assimilation of NH_4_^+^ derived from photorespiration (Lancien *et al*. 2002; Konishi *et al*. 2014).

The lack of malate valve capacities leads to improved growth when *nadp‐mdh* or *plnad‐mdh/plNAD‐MDH Arabidopsis* mutants are grown on nitrate or on ammonium, respectively, as the sole N source (Hebbelmann *et al*. 2012; Selinski *et al*. 2014). Under these conditions, Fd‐GOGAT transcripts are increased in *nadp‐mdh* knockout plants, and NADH‐GOGAT transcripts are more abundant in heterozygous *plnad‐mdh/plNAD‐MDH* mutants (Selinski & Scheibe 2014). In the case of *nadp‐mdh* knockout mutants, nitrite reductase as part of nitrate assimilation and Fd‐GOGAT which contributes to the assimilation of NH_4_^+^ derived from photorespiration, serve as alternative electron acceptors for reduced Fd and increase nitrate assimilation, thereby preventing, at the same time, the accumulation of excess NADPH (Hebbelmann *et al*. 2012; Selinski & Scheibe 2014). Correspondingly, when the dark malate valve capacity is decreased, NADH‐GOGAT, as a key enzyme of NH_4_^+^ assimilation, serves as an alternative mechanism maintaining redox homeostasis by removing NADH generated by GapCp during plastidial glycolysis (Fig. 4; Selinski *et al*. 2014; Selinski & Scheibe 2014).

Measurements of mitochondrial respiration in spinach leaves indicate that NADH formed inside mitochondria is oxidised by a malate/OAA shuttle to serve extra‐mitochondrial processes, *e.g*. reduction of nitrate in the cytosol or of hydroxypyruvate in the peroxisomes (Hanning & Heldt 1993). Furthermore, malate oxidation was shown to be one of the major sources of NADH for nitrate reduction in corn leaf blades (Neyra & Hageman 1976) that is catalysed by cytosolic NAD‐MDH isoform(s).

The malate/OAA shuttle, which itself is reversible depending on the concentrations of OAA and malate on both sides of the membrane, functions as a ‘thermodynamic buffer’ that maintains the chloroplast NADP^+^/NADPH ratio constant over a wide range of conditions. This corresponds to the concept put forward for the adenylate ratio for the first time by Stucki (1980). Malate valves are, therefore, important to communicate between cellular compartments and to optimise photosynthesis (Raghavendra & Padmasree 2003).

## Conclusion

Malate valves, consisting of malate dehydrogenases and dicarboxylate transporters, are essential features for balancing energy metabolism and maintaining redox homeostasis in plants. In combination, they serve as thermodynamic buffers. The presence of different MDH isoforms in various subcellular compartments and their distinct roles enable plants to adapt to various stress conditions as well as to control growth and development. Although there has been much progress in unravelling roles and regulation of MDH isoforms in plants in recent years, knowledge about detailed mechanisms and controlled interplay between the systems at the various regulatory levels is still lacking in some cases.
